# Effect of Intermittent Theta-Burst Stimulation on Chronobiological Hypothalamic-Pituitary-Thyroid Axis Activity in Treatment-Resistant Depression

**DOI:** 10.1192/j.eurpsy.2026.10718

**Published:** 2026-07-31

**Authors:** F. Duval, M.-C. Mokrani, V. Danila, T. Weiss, F. Gonzalez Lopera, M. I. Tomsa

**Affiliations:** Pole 8/9 - APF2R, Centre Hospitalier, Rouffach, France

## Abstract

**Introduction:**

The effects of intermittent theta-burst stimulation (iTBS), a form of repetitive transcranial magnetic stimulation, on the hypothalamic-pituitary-thyroid (HPT) axis are not well understood. This axis is frequently dysregulated in treatment-resistant depression (TRD). We previously demonstrated that the difference in thyrotropin (TSH) response to protirelin (TRH) between 23 h and 8 h on the same day (the ∆∆TSH test) is a highly sensitive chronobiological marker.

**Objectives:**

To evaluate the impact of iTBS targeting the left dorsolateral prefrontal cortex (LDPFC) on chronobiological HPT activity in hospitalized TRD patients.

**Methods:**

Twenty-three TRD inpatients with abnormal ∆∆TSH values (< 2.5 mU/L) and HAMD-17 scores ≥ 18 were compared to 23 matched healthy controls (HCs). Patients received 20 iTBS sessions (one per weekday over four weeks) while maintaining stable antidepressant treatment. The ∆∆TSH test and HAMD-17 scores were assessed pre- and post-treatment. Response was defined as a ≥ 50% reduction in HAMD-17.

**Results:**

Baseline ∆∆TSH values were significantly lower in TRDs than in HCs (p < 0.00001, by U test). After iTBS treatment, patients showed a decrease in HAMD-17 scores (p = 0.0001, by T test) and an increase in ∆∆TSH values (p = 0.001, by T test)—although still remaining lower than those of HCs (p = 0.03, by U test). At endpoint, HAMD-17 scores correlated negatively with ∆∆TSH values (rho = -0.74, p = 0.0004). Fourteen patients (61%) normalized their ∆∆TSH, of whom 11 (78%) were responders; all patients who did not normalize their post-treatment ∆∆TSH (n = 9) were non-responders (p = 0.0003, by Fisher’s Exact test).

**Conclusions:**

While the underlying mechanisms remain to be clarified, this pilot study suggests that successful iTBS treatment may help restore normal chronobiological function of the HPT axis in TRD patients, and that such restoration is associated with clinical improvement.

**Disclosure of Interest:**

None Declared

